# 供者来源CAR-T细胞治疗异基因造血干细胞移植后复发急性B淋巴细胞白血病的疗效及安全性

**DOI:** 10.3760/cma.j.cn121090-20230815-00068

**Published:** 2024-01

**Authors:** 亚琪 卓, 三芳 涂, 璇 周, 继龙 杨, 丽娟 周, 睿 黄, 宇贤 黄, 梅芳 李, 波 金, 博 王, 诗琦 黎, 忠涛 袁, 丽华 张, 林 刘, 三斌 王, 玉华 李

**Affiliations:** 1 南方医科大学珠江医院血液科，广州 510280 Department of Hematology, Zhujiang Hospital, Southern Medical University, Guangzhou 510280, China; 2 解放军联勤保障部队第九二〇医院血液科，昆明 650118 Department of Hematology, 920th Hospital of Joint Logistics Support Force of PLA, Kunming 650118, China

**Keywords:** 供者来源, 嵌合抗原受体, 异基因造血干细胞移植, 急性B淋巴细胞白血病, 复发, Donor-derived, Chimeric antigen receptor, Allogeneic hematopoietic stem cell transplantation, B-cell acute lymphoblastic leukemia, Relapsed

## Abstract

**目的:**

探索供者来源靶向CD19或联合靶向CD22嵌合抗原受体T细胞（CAR-T细胞）治疗异基因造血干细胞移植后复发急性B淋巴细胞白血病（B-ALL）的有效性及安全性。

**方法:**

回顾性分析2015年9月至2022年12月在南方医科大学珠江医院及解放军联勤保障部队第九二〇医院血液科22例接受供者来源 CAR-T细胞治疗的异基因造血干细胞移植后复发的B-ALL患者的有效性和安全性。主要研究终点是总生存（OS），次要研究终点是无事件生存（EFS）、完全缓解（CR）率、3～4级不良事件。

**结果:**

CAR-T细胞输注后，18例（81.82％）患者获得CR且微小残留病（MRD）均为阴性。中位随访1037（95％*CI* 546～1509）d，中位OS期为287（95％*CI* 132～441）d，中位EFS期为212（95％*CI* 120～303）d，6个月OS率为67.90％（95％*CI* 48.30％～84.50％），6个月EFS率为58.70％（95％*CI* 37.92％～79.48％），1年OS率为41.10％（95％*CI* 19.15％～63.05％），1年EFS率为34.30％（95％*CI* 13.92％～54.68％）。3例（13.64％）患者发生≥3级细胞因子释放综合征（CRS），未发生免疫效应细胞相关神经毒性综合征（ICANS），2例患者发生急性移植物抗宿主病（aGVHD）（分别为Ⅱ度和Ⅳ度）。

**结论:**

供者来源CAR-T细胞治疗异基因造血干细胞移植后复发B-ALL是一种安全有效的治疗策略。

## Abstract (en)

**Objective:**

To investigated the safety and efficacy of donor-derived CD19+ or sequential CD19+ CD22+ chimeric antigen receptor T-cell (CAR-T) therapy in patients with B-cell acute lymphoblastic leukemia (B-ALL) after allogeneic hematopoietic stem cell transplantation (allo-HSCT).

**Methods:**

The data of 22 patients with B-ALL who relapsed after allo-HSCT and who underwent donor-derived CAR-T therapy at the Zhujiang Hospital of Southern Medical University and the 920th Hospital of Joint Logistics Support Force of the People's Liberation Army of China from September 2015 to December 2022 were retrospectively analyzed. The primary endpoint was overall survival (OS), and the secondary endpoints were event-free survival (EFS), complete remission (CR) rate, and Grade 3−4 adverse events.

**Results:**

A total of 81.82% (*n*＝18) of the 22 patients achieved minimal residual disease-negative CR after CAR-T infusion. The median follow-up time was 1037 (95% *CI* 546–1509) days, and the median OS and EFS were 287 (95% *CI* 132−441) days and 212 (95% *CI* 120−303) days, respectively. The 6-month OS and EFS rates were 67.90% (95% *CI* 48.30%−84.50%) and 58.70% (95% *CI* 37.92%−79.48%), respectively, and the 1-year OS and EFS rates were 41.10% (95% *CI* 19.15%−63.05%) and 34.30% (95% *CI* 13.92%−54.68%), respectively. Grade 1−2 cytokine release syndrome occurred in 36.36% (*n*＝8) of the patients, and grade 3−4 occurred in 13.64% of the patients (*n*＝3). Grade 2 and 4 graft-versus-host disease occurred in two patients.

**Conclusion:**

Donor-derived CAR-T therapy is safe and effective in patients with relapsed B-ALL after allo-HSCT.

异基因造血干细胞移植（allo-HSCT）是急性淋巴细胞白血病（ALL）一种重要的治疗手段[1]，但allo-HSCT后仍有20％～40％患者复发。移植后复发治疗选择非常有限[2]–[3]，挽救性化疗、供者淋巴细胞输注（DLI）、二次移植等传统方案的治疗反应及远期生存不容乐观[4]。免疫治疗包括嵌合抗原受体T细胞（CAR-T细胞）、双特异性抗体、抗体偶联药物等正逐渐提高移植后复发患者的疗效[5]。CD19 CAR-T细胞治疗在复发/难治急性B淋巴细胞白血病（B-ALL）患者中的完全缓解（CR）率可达80％[6]–[7]，但移植后复发患者存在T细胞采集时机受限、数量不足、功能差、增殖能力有限、体内持续时间短等局限[8]。一个潜在方法是采集供者来源的T细胞进行CAR-T细胞的制备。而供者来源CAR-T细胞的疗效、在体内的持续时间、扩增水平、免疫毒性及急性移植物抗宿主病（aGVHD）等目前处于研究探索阶段，尚未见大样本报道。本研究中，我们回顾性分析供者来源CAR-T细胞治疗allo-HSCT后复发B-ALL的有效性和安全性，现报道如下。

## 病例与方法

1. 病例资料：回顾性分析2015年9月至2022年12月在南方医科大学珠江医院及解放军联勤保障部队920医院血液科接受供者来源CAR-T细胞治疗allo-HSCT后复发的22例 B-ALL患者临床资料。

2. CAR-T细胞治疗：利用血细胞分离机采集供者外周血单个核细胞，分选出外周血CD3^+^ T细胞，用编码CAR的慢病毒载体转染T细胞，制备靶向CD19或CD22 CAR-T细胞，15例为鼠源CAR-T，共刺激域为CD28（来源于珠江医院）；7例为人源化CAR-T，共刺激域为4-1BB（来源于920医院）。19例患者在CAR-T细胞回输前行常规FC方案（氟达拉滨 30 mg·m^−2^·d^−1^×3 d，环磷酰胺 300 mg·m^−2^·d^−1^×3 d）预处理，1例患者行FCD方案（氟达拉滨 30 mg·m^−2^·d^−1^×3 d，环磷酰胺 300 mg·m^−2^·d^−1^×3 d，地塞米松25 mg/d×3 d）预处理，2例因身体状况差未行预处理。预处理结束后1～2 d进行CAR-T细胞回输，回输当天为第0天。其中有16例接受CD19 CAR-T细胞回输，6例接受CD19联合CD22 CAR-T细胞回输。CD19 CAR-T细胞中位回输量为2.31（0.08～8.28）×10^6^/kg，CD22 CAR-T细胞中位回输量为2.82（2.12～8.18）×10^6^/kg。接受靶向CD19联合CD22 CAR-T细胞回输的患者采用续贯输注方式，中位间隔时间为7（0～54）d。

3. 疗效及不良反应评估：疗效评估参考《中国成人急性淋巴细胞白血病诊断与治疗指南（2021年版）》[9]；流式细胞术检测微小残留病（MRD）水平，MRD<0.01％为阴性[10]，MRD≥30％定义为高肿瘤负荷。总生存（OS）期定义为首次输注CAR-T到随访终点或死亡的间隔时间；无事件生存（EFS）期定义为患者接受首次CAR-T输注到首次复发、随访终点或死亡的间隔时间。细胞因子释放综合征（CRS）参照Lee等的分级标准进行评估与分级[11]，免疫效应细胞相关神经毒性综合征（ICANS）的分级参照CARTOX标准。aGVHD的诊断和分级按aGVHD国际联盟（MAGIC）分级标准[12]；根据美国国立卫生研究院（NIH）慢性移植物抗宿主病（cGVHD）的分级评分系统[13]对cGVHD进行严重程度分级。

4. 统计学处理：使用SPSS 26.0软件进行数据分析。连续变量以*M*（范围）进行统计描述，分类变量以例数（构成比）进行统计描述。采用Kaplan-Meier法绘制生存曲线，两组间比较采用Log-rank检验。双侧*P*<0.05为差异有统计学意义。

## 结果

一、患者基线特征

22例患者中，男11例，女11例，中位年龄28.5（9～43）岁，移植类型：亲缘全相合移植12例（54.5％），单倍体造血干细胞移植10例（45.5％）。其中13例（59.1％）患者为移植后早期复发；10例（45.4％）具有高危细胞/分子遗传学改变（包括MLL-AF4融合基因阳性、BCR-ABL融合基因阳性、WT1突变等）；4例在CAR-T输注前处于高肿瘤负荷状态；3例发生髓外侵犯（1例侵犯膀胱、附件区以及腹腔，2例侵犯中枢神经系统）。18例患者在CAR-T输注前供者细胞嵌合度为完全嵌合，4例为混合嵌合；美国东部肿瘤协作组体力状态（ECOG）评分中位数为3（1～4）分。患者临床结局详见图1。

**图1:**
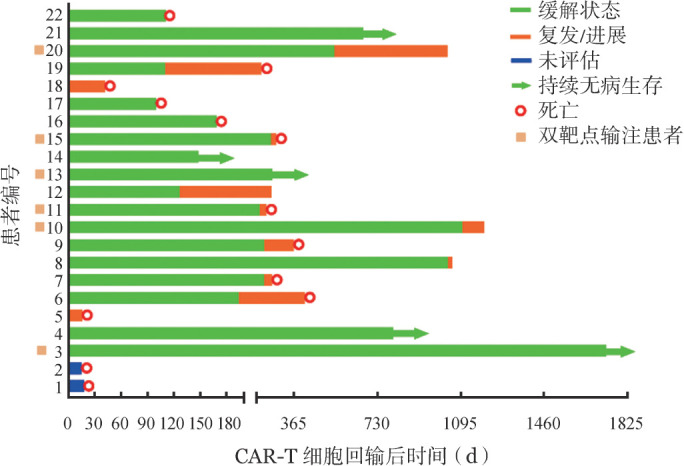
22例allo-HSCT后复发B-ALL患者CAR-T治疗后临床结局 注 CAR-T细胞：嵌合抗原受体T细胞；allo-HSCT：异基因造血干细胞移植；B-ALL：急性B淋巴细胞白血病

二、CAR-T细胞输注后疗效评估

接受CAR-T细胞治疗的22例患者中18例（81.8％）在CAR-T输注后1个月获得MRD阴性CR，2例因疾病持续进展，1例于第18天因感染死亡无法评估，1例因重度CRS及aGVHD死亡无法评估。在18例输注CAR-T后获得CR的患者中，13例（72.2％）出现复发，中位复发时间为798（95％*CI* 166～1429）d。

三、CAR-T细胞输注后生存分析

中位随访1037（95％*CI* 546～1509）d，5例（27.8％）长期无病生存，2例因疾病持续进展死亡，复发患者中9例均因疾病进展死亡，另外4例通过二次移植、挽救化疗或免疫治疗，获得长期生存。中位OS期为287（95％*CI* 132～441）d，中位EFS期为212（95％*CI* 120～303）d，6个月OS率为67.90％（95％*CI* 48.30％～84.50％），6个月EFS率为58.70％（95％*CI* 37.92％～79.48％），1年OS率为41.10％（95％*CI* 19.15％～63.05％），1年EFS率为34.30％（95％*CI* 13.92％～54.68％）。移植后早期复发与晚期复发、CAR-T输注前MRD阳性与阴性患者的OS、EFS差异无统计学意义；而伴髓外侵犯的患者OS、EFS率更低；未发生CRS患者的OS、EFS率高于发生CRS患者，获得MRD（阴性）CR的患者OS、EFS率更高，CAR-T输注前供者细胞嵌合状态为完全嵌合的患者OS、EFS率更高（图2）。

**图2:**
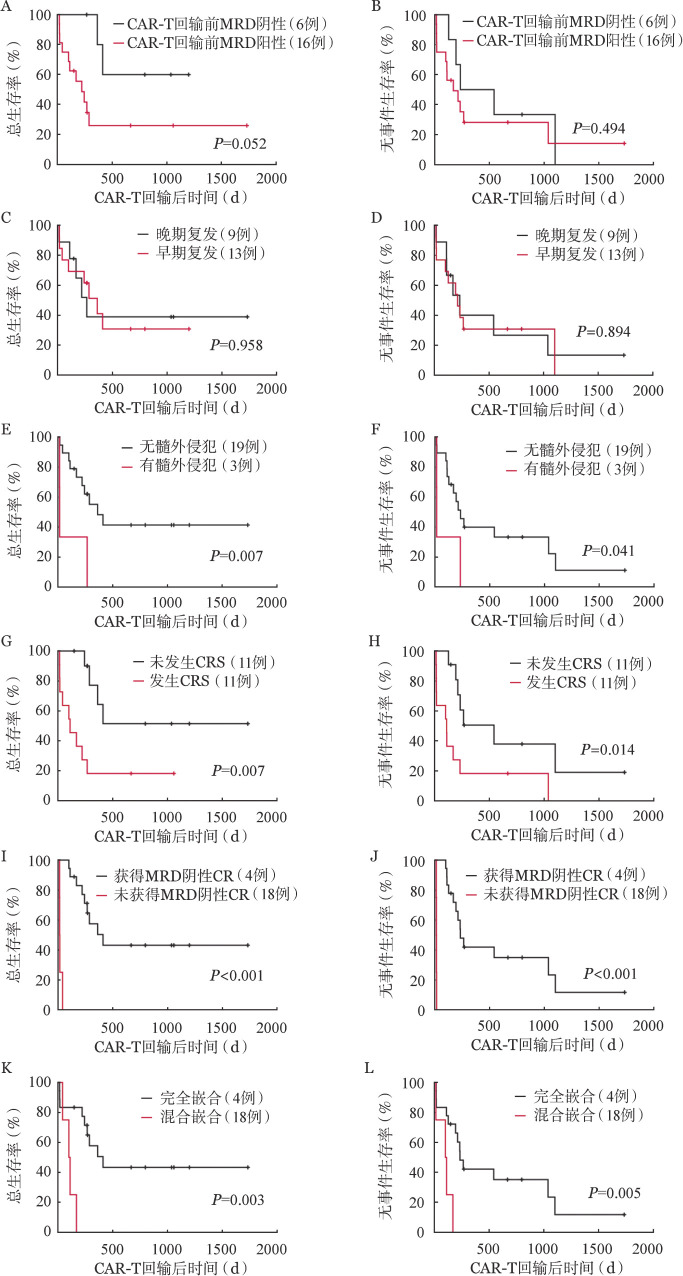
22例接受供者来源CAR-T细胞治疗allo-HSCT后复发的急性B淋巴细胞白血病（B-ALL）患者生存曲线 **A**、**B** CAR-T回输前MRD阴性与阳性组；**C**、**D**移植后早期复发（<6个月）与晚期复发（≥6个月）组；**E**、**F** CAR-T前有无髓外侵犯组；**G**、**H**是否发生CRS组；**I**、**J** CAR-T后是否获得MRD阴性CR组；**K**、**L** CAR-T回输前供者细胞嵌合状态为完全嵌合及混合嵌合组 注 CAR-T细胞：嵌合抗原受体T细胞；allo-HSCT：异基因造血干细胞移植；MRD：微小残留病；CRS：细胞因子释放综合征；CR：完全缓解

四、CAR-T细胞输注后不良反应评估

22例接受CAR-T细胞输注的患者中，共11例（50.0％）发生CRS，中位发生时间为+7（+3～+10）d，中位持续5（2～12）d，IL-6中位达峰时间为+7（+1～+17）d。8例（36.36％）患者发生的CRS为1～2级，主要症状为高热、关节疼痛，2例予托珠单抗治疗后缓解，所有患者均进行退热对症处理；2例（9.09％）患者发生3级CRS，主要症状为高热、低血压、低血氧，予地塞米松联合芦可替尼治疗，分别持续3、10 d后获得控制；1例（4.55％，例2）患者+5 d发生4级CRS，主要症状为持续高热、低血压，IL-6水平急剧升高，先后予退热、托珠单抗（320 mg×2次）、补液、升压、地塞米松（总量120 mg）治疗，于+15 d死亡。严重CRS（≥3级）发生率为13.64％，发生严重CRS的患者输注前均为高肿瘤负荷。所有患者均未发生ICANS。2例患者发生血流感染，其中1例死亡。

共2例患者发生aGVHD，值得注意的是，例2在2018年移植后发生Ⅲ度aGVHD（肝脏、肠道），先后予环孢素A联合甲泼尼龙、巴利昔单抗、间充质干细胞抗GVHD治疗后达CR；于移植后第110天发生Ⅲ度cGVHD（肠道、皮肤），持续环孢素A抗GVHD治疗后部分缓解；移植后第204天出现骨髓复发，经挽救性化疗后仍持续进展，遂纳入CAR-T临床试验，−7 d停用环孢素A，FC方案预处理后出现粒细胞缺乏伴发热，予抗感染治疗，体温逐渐控制；+5 d出现4级CRS，+8 d出现严重腹痛、腹泻、胆红素进行性升高，诊断Ⅳ度aGVHD（肠道、肝脏），予环孢素A联合甲泼尼龙、芦可替尼抗GVHD治疗，+15 d治疗无效死亡。另1例（例15）发生Ⅱ度aGVHD（肠道、皮肤、肝脏），予泼尼松、西罗莫司抗GVHD治疗后控制。CAR-T治疗后所有患者均未出现cGVHD。具体CAR-T相关不良反应见表1。

**表1:** 22例接受供者来源CAR-T细胞治疗allo-HSCT后复发的B-ALL患者基线特征、CAR-T靶点及输注量、疗效及不良反应

治疗中心	例号	性别	年龄（岁）	细胞/分子遗传学改变	入组前MRD（%）	预处理前桥接治疗方案	CAR-T输注前MRD（%）	CAR-T靶点	输注剂量（×10^6^/kg）	输注后1个月内疗效	CRS分级	aGVHD分度
ZH	1	女	43	无	42.0	地西他滨+CAG	37.0	CD19	2.21	NA	1	无
ZH	2	女	19	无	48.6	无	NA	CD19	2.82	NA	4	Ⅳ
ZH	3 ^a^	男	19	Ph (+)	14.5	DLI	25.0	CD19/CD22	4.63	MRD(−)	无	无
ZH	4	男	29	无	6.8	VTP	<0.01	CD19	2.63	MRD(−)	无	无
ZH	5 ^b^	女	28	无	<0.01	MA	<0.01	CD19	2.07	PD	2	无
ZH	6	女	32	无	66.8	VTLP	<0.01	CD19	3.02	MRD(−)	无	无
ZH	7	男	32	无	40.0	VMCP	3.0	CD19	1.80	MRD(−)	1	无
ZH	8	男	34	MLL-AF4	26.9	VTCP	17.4	CD19	2.50	MRD(−)	1	无
ZH	9	男	41	WT1、PAX5	45.0	VDP	<0.01	CD19	1.06	MRD(−)	无	无
ZH	10	女	25	Ph (+)、WT1	70.5	VCP	<0.01	CD19/CD22	3.13/2.12	MRD(−)	无	无
ZH	11	女	22	复杂核型	71.8	VP	26.9	CD19/CD22	2.30/2.60	MRD(−)	无	无
ZH	12	女	20	无	78.0	VP+贝林妥欧单抗	<0.01	CD19	7.94	MRD(−)	无	无
ZH	13	男	25	Ph (+)	0.5	无	0.3	CD19/CD22	8.22/8.18	MRD(−)	无	无
ZH	14	女	32	Ph (+)	65.2	VCP	5.1	CD19	4.46	MRD(−)	无	无
ZH	15	女	42	MLL-AF4	61.8	hyper-CVAD A	84.8	CD19/CD22	2.54/3.03	MRD(−)	无	Ⅱ
920	16	男	11	E2A/PBX	63.0	无	1.4	CD19	1.88	MRD(−)	1	无
920	17	男	22	无	58.0	无	76.4	CD19	1.72	MRD(−)	3	无
920	18	女	30	Ph (+)	90.0	无	11.9	CD19	2.06	PD	3	无
920	19	女	32	无	21.0	无	0.1	CD19	2.32	MRD(−)	1	无
920	20 ^a^	男	32	无	8.0	无	<0.01	CD19/CD22	2.66	MRD(−)	无	无
920	21	男	17	无	23.0	无	1.4	CD19	0.0896	MRD(−)	1	无
920	22	男	9	无	55.0	无	17.5	CD19	0.0824	MRD(−)	1	无

注 CAR-T细胞：嵌合抗原受体T细胞；allo-HSCT：异基因造血干细胞移植；ZH：珠江医院；920：解放军联勤保障部队第九二〇医院；CAG：吡柔比星+阿糖胞苷+G-CSF；DLI：供者淋巴细胞输注；VTP：长春新碱+吡柔比星+地塞米松；MA：米托蒽醌+阿糖胞苷；VTLP：长春新碱+吡柔比星+左旋门冬酰胺酶+地塞米松；VMCP：长春新碱+米托蒽醌+环磷酰胺+地塞米松；VTCP：长春新碱+吡柔比星+环磷酰胺+地塞米松；VDP：长春新碱+伊达比星+地塞米松；VCP：长春新碱+环磷酰胺+地塞米松；VP：长春新碱+地塞米松；hyper-CVAD A：环磷酰胺+长春新碱+多柔比星+地塞米松；MRD：微小残留病；PD：疾病进展；CRS：细胞因子释放综合征；aGVHD：急性移植物抗宿主病；NA：未评估。^a^ CAR-T输注量为CD19、CD22 CAR-T细胞数之和；^b^入组前及CAR-T输注前仅髓外侵犯

五、CAR-T细胞扩增

22例患者体内CAR-T细胞中位达峰时间为+11（+6～+48）d，其中12例患者持续监测CAR-T细胞数，体内CAR-T细胞中位持续时间为184（13～810）d。

## 讨论

相较于移植后复发患者的传统治疗方案，例如化疗、DLI等，接受供者来源CAR-T细胞治疗患者的疗效显著改善[14]–[15]，表2中的研究结果显示接受供者来源CAR-T治疗患者的CR率超过均60％。本研究22例患者中81.8％获得MRD阴性CR，中位OS期为287（95％*CI* 132～441）d，中位EFS期为212（95％*CI* 120～303）d。提示，对于allo-HSCT后复发的B-ALL患者，输注供者CAR-T细胞可能是一种有效的策略。多项研究显示，移植后早期复发、GVHD的发生是预后不良的因素[18]–[19]，而本研究中allo-HSCT后早期复发患者及晚期复发患者的预后较差，OS、EFS差异无统计学意义，提示可能移植后晚期复发的患者获益有限。研究表明，髓外侵犯患者可以从CAR-T细胞治疗中获益，但CR率仍低于无髓外侵犯患者，且获得治疗反应的时间滞后于骨髓治疗反应的时间，这可能与髓外侵犯中CAR-T细胞运输和衰竭机制相关。除此之外，多灶性髓外侵犯患者获得CR更难，预后较单部位髓外侵犯患者更差[20]–[21]。本研究3例髓外侵犯患者中，1例（例2）死于CAR-T相关不良事件，1例疾病持续进展后死亡，仅1例获得MRD阴性CR，但于输注后8个月复发死亡，且髓外侵犯患者的OS、EFS率更低。这提示对于髓外侵犯患者，单用供者来源CAR-T细胞治疗可能很难获得长期生存。

**表2:** 供者来源CAR-T细胞治疗allo-HSCT后复发B-ALL患者临床数据

参考文献	例数	靶点	CR率（%）	预后情况	GVHD发生率（%）	CRS发生率（%）	ICANS发生率（%）
Tan等[14]	22	CD19	72.7	1年LFS率54.5%（95%*CI* 19.6%~39.8%）；1年OS率59.1%（95% *CI* 22.1%~41.9%）	9.1	86.4	9.1
Hua等[15]	13	CD19	61.5	中位CRD 8（95% *CI* 3~25）个月；中位OS期9.5（95% *CI* 3~25）个月	7.7	23.1	0
Hua等[16]	11	CD19	63.6	中位CRD 8（95% *CI* 1~22）个月；中位OS期9（95% *CI* 2~22）个月	4.5	54.5	0
马润芝等[17]	9	CD19	100.0	中位LFS期18.1（95% *CI* 3.7~35.7）个月	11.0	100.0	44.0

注 CAR-T细胞：嵌合抗原受体T细胞；allo-HSCT：异基因造血干细胞移植；B-ALL：急性B淋巴细胞白血病；CR：完全缓解；GVHD：移植物抗宿主病；CRS：细胞因子释放综合征；ICANS：免疫效应细胞相关神经毒性综合征；PFS：无进展生存；OS：总生存；LFS：无白血病生存；CRD：完全缓解期

国内外研究显示供者来源CAR-T细胞有显著的抗白血病作用及良好的安全性[22]。本研究中11例（50.0％）患者发生CRS，仅3例（13.6％）出现严重CRS，无发生ICANS的患者，提示对于allo-HSCT后复发的B-ALL患者，输注供者CAR-T细胞具有良好安全性。而CRS等不良反应严重程度与肿瘤负荷、既往治疗方案、疾病类型、CAR-T类型及剂量等相关[23]–[24]，需要进行有效的管理及预防，研究显示可以通过CAR-T前的桥接治疗、高强度预处理等方案来降低高肿瘤负荷从而有效预防不良反应的发生同时提高CAR-T疗效[25]–[26]。

值得注意的是，除了CRS和ICANS等并发症外，异基因CAR-T细胞输注也可能增加GVHD发生风险。迄今为止，关于CAR-T细胞治疗移植后复发B-ALL患者的数据显示，GVHD的发生率较低[22],[27]–[28]，在124例allo-HSCT后复发接受DLI或CAR-T治疗的患者中，DLI相关aGVHD的累积发生率明显高于CAR-T组[29]，但异基因CAR-T细胞相关性GVHD的危险因素尚未完全确定，可能与T细胞的来源、CAR结构、CAR-T细胞亚群、allo-HSCT后GVHD的病史有关[20],[22],[30]。一项研究中，23％的患者发生了CAR-T相关GVHD，并与CAR-T前存在cGVHD具有相关性，但也有研究提示CAR-T细胞输注后的新发GVHD与allo-HSCT后的GVHD无明显相关性，这表明CAR-T治疗对既往发生过GVHD的患者也可能是一种安全的治疗选择[18]，本研究中仅2例患者发生aGVHD，无发生cGVHD的患者，GVHD发生率较低，但值得注意的是例2在移植相关cGVHD尚未完全控制时出现疾病复发，并于-7 d才停用免疫抑制剂，输注CAR-T后出现Ⅳ度aGVHD，这提示对于同时存在活动性GVHD的患者，接受CAR-T细胞治疗后发生CAR-T相关 GVHD的风险可能增加。因此，EBMT指南也明确指出allo-HSCT后接受CAR-T治疗需满足无活动性GVHD且停用免疫抑制剂1个月后方可进行[31]。供者来源CAR-T细胞治疗相关GVHD发生率较低的机制也在逐步阐明，一方面，GVHD的发生与输注淋巴细胞数量有关，T 细胞的数量未达到GVHD发生的阈值；另一方面，由于双特异性TCR和CAR信号通路发生累积过度激活、PD-1在供者来源的CAR-T细胞中的表达增加[27]，导致T细胞功能下降以及耗竭增多，从而降低GVHD发生率，但仍然保留较强的抗肿瘤效应[32]。尽管大多数GVHD症状较轻，但供者来源CAR-T治疗相关GVHD仍然需要被重视。

CAR-T细胞治疗尽管可获得较高缓解率，但仍有约半数患者在治疗后出现疾病复发，这是目前面临的重要挑战。CD19抗原的丢失、T细胞功能的下降及持久性有限、高危遗传学改变、多线治疗的耐药以及抑制性免疫微环境等因素会导致自体来源CD19 CAR-T细胞免疫治疗后的复发和无反应[33]–[34]，而异基因CAR-T细胞治疗也存在同样的挑战。研究显示，异基因和自体CAR-T细胞均表现为效应T细胞的早期扩增，但异基因CAR-T细胞有较高的峰值[35]以及更高水平的PD-1表达[27]，这些因素都加速细胞耗竭，导致细胞持久性有限，这可能是复发的主要机制之一。截至随访终点，18例输注CAR-T后获得CR的患者中13例（72.2％）复发，其中9例死亡，4例长期生存并接受其他治疗。由此可见，CAR-T治疗后复发仍是影响患者长期生存的重要因素。CAR-T联合分子靶向治疗、免疫检查点抑制剂或多靶点CAR-T输注是否可降低复发率值得进一步探索[18]。虽然有较多临床数据报道了CAR-T细胞治疗allo-HSCT后复发B-ALL的病例，但CAR-T输注前后供者细胞嵌合数据分析甚少。本研究中4例在CAR-T前供者嵌合状态为混合嵌合的患者最终都出现疾病复发并且死亡，完全嵌合组OS、EFS期长于混合嵌合组，提示allo-HSCT后复发的B-ALL患者在输注供者来源CAR-T 细胞前供者细胞嵌合度越高，CR率越高，生存期越长。因此，桥接及预处理化疗非常重要，其在增强供者T细胞的植入、T细胞持久性并提高疗效中发挥重要作用[36]–[37]。本研究中所有患者allo-HSCT后的复发均为受者来源复发，且复发时均存在供者细胞嵌合度下降，其中13例患者在预处理前行桥接治疗，降低了回输前肿瘤负荷的同时也使这些患者再次获得供者细胞完全嵌合，这使供者来源CAR-T细胞更好地发挥功能且不被受者排异。

总之，本研究显示供者来源CAR-T细胞治疗allo-HSCT后复发的B-ALL患者具有显著的缓解率和较长时间生存期，且严重CRS、ICANS以及GVHD等不良反应发生率较低，提示供者来源CAR-T细胞治疗allo-HSCT后复发的B-ALL是一种安全有效的策略。本研究仍存在一些局限性，如回顾性分析、样本量较少；因此，需要更多大规模前瞻性多中心临床试验来进一步探索，并逐步明确潜在机制。
